# No evidence for quorum sensing during egg hatching in the cestode *Schistocephalus solidus*

**DOI:** 10.7717/peerj.20667

**Published:** 2026-02-03

**Authors:** Emily V. Kerns, Sara Engel, Panna A. Codner, Jesse N. Weber

**Affiliations:** Department of Integrative Biology, University of Wisconsin-Madison, Madison, WI, United States of America

**Keywords:** *Schistocephalus solidus*, Threespine stickleback, Quorum sensing, Cestoda, Coinfection, Inbreeding depression

## Abstract

*Schistocephalus solidus*, a parasitic cestode with a multi-host life cycle, reproduces in its terminal host either by outcrossing with similarly sized individuals or selfing. Previous work found that selfing greatly depresses egg hatching rates, presumably as a result of inbreeding depression. We designed an experiment to test whether *S. solidus* evolved quorum sensing (QS) during hatching as a mechanism to facilitate synchronized infection, thereby increasing the opportunity for outcrossing in its terminal host. We also performed exploratory analyses to test whether QS varies across parasite populations and cross type (*i.e.*, whether progeny were produced *via* outcrossing or selfing), though these had limited statistical power due to low sample sizes across treatments. We predicted that if QS was present, it would be common across all populations and that higher egg density within a small area would result in higher hatching rates. We also expected that outcrossed eggs would hatch at higher rates than those produced *via* selfing. While we found different hatching rates between populations, there was no evidence for QS. We also observed that selfed eggs hatched at lower rates than outcrossed eggs, replicating previous findings. Although we failed to find density dependent hatching within the scope of our sample size, we discuss the conditions that may either favor or disfavor QS evolution across *S. solidus* and other helminth populations.

## Introduction

Despite their ubiquity and diversity, parasites are a historically understudied group of organisms. Although most research focuses on host biology, parasites are a strong force of natural selection that can drive drastic demographic shifts and heritable genetic changes in hosts over ecological time scales (Gowler et al., 2023; Ling et al., 2020; Rogowski et al., 2020). For example, helminths often infect small hosts during their early stages of development and exploit large, high trophic level hosts at later developmental stages to optimize both transmission and growth (Benesh, 2023; Parker, Ball & Chubb, 2015). Thus, the ecological and evolutionary effects of a single helminth species can span across multiple trophic levels. A long-standing model system for studying the evolution and ecology of parasitism is the threespine stickleback (*Gasterosteus aculeatus*) fish and its parasitic cestode *Schistocephalus solidus* (Barber, 2013). While *S. solidus* clearly imposes strong selection on stickleback to mount various forms of resistance and tolerance (Weber et al., 2022), the cestode has received much less research attention than its fish host, particularly during its early life stages.

*S. solidus* was the first reported parasite with a complex, trophically transmitted life cycle (Abildgaard, 1790; Barber, 2013). Initially, the cestode’s eggs develop in freshwater before hatching into coracidia (Barber, Berkhout & Ismail, 2016). The coracidia are ingested by its first intermediate host, copepods in the order Cyclopoida, and then migrate into the host body cavity before developing into procercoid larvae. For the parasite’s life cycle to progress, infected copepods must be ingested by threespine stickleback, the specific host for *S. solidus*. *S. solidus* migrates from the gut into the stickleback’s body cavity, where the majority of the cestode’s growth occurs as a plerocercoid larva (Barber, 2013). The infected fish is then predated on by a bird, *S. solidus’*s terminal host, where the parasite does not siphon any resources but rather immediately develops its reproductive anatomy within the first few days of entering the bird gut (Hopkins & Smyth, 1951; Smyth, 1946). For 5–10 days following ingestion, fertilized eggs are generated *via* either cross fertilization or selfing, and are then expelled *via* bird feces. The life history of *S. solidus* is relatively uncommon, compared to other cestodes, in that its specific host (threespine stickleback) is not its definitive host. Because its final bird host can travel large distances while carrying the parasite, there is little evidence of genetic differentiation among *S. solidus* in nearby lakes, though signatures of isolation-by-distance do appear over larger geographic scales (Sprehn et al., 2015; Strobel et al., 2016).

Inbreeding depression is extremely costly for *S. solidus*, with progeny of selfed individuals exhibiting less than 10% of the hatching rate of their outcrossed counterparts (Benesh et al., 2014). The outcrossing potential for this cestode depends not only on whether an individual encounters another mature parasite of similar size inside the gut of its bird host, but also on both parasites being consumed at approximately the same time to coordinate the exchange of gametes. *S. solidus* coinfections of threespine stickleback are common, which increases both the probability of finding a mate and synchronizing the onset of breeding. However, there is a crowding effect in both stickleback and copepods in which coinfection decreases cestode size, resulting in reduced fecundity compared to *S. solidus* found in singly infected hosts (Heins, Baker & Martin, 2002; Weber et al., 2017; Wedekind, 1997). Yet, hatched *S. solidus* coracida are more likely to successfully establish infection in copepods when exposure rates are high (Wedekind, 1997). Although the costs and benefits of synchronized infection may vary across the *S. solidus* life cycle, it is plausible that any traits facilitating coinfection of similar-sized parasites in terminal hosts would be strongly favored given the large fitness differences between producing selfed *vs* outcrossed progeny. 

One mechanism that could facilitate synchronous infections and outcrossing in *S. solidus* is quorum sensing (QS) during egg hatching. First described in bacterial cells, QS involves sensing local population density and then releasing a signal when a critical density is reached (Waters & Bassler, 2005). While typically associated with bacteria, the eukaryotic parasite *Trypanosoma brucei*, photosynthetic protists *Chlamydomonas reinhardtii* and *C. moewusii*, and the model organism *Saccharomyces cerevisiae* have been shown to exhibit QS properties (Chen & Fink, 2006; Folcik et al., 2020; Matthews, 2021). These studies not only document the presence of QS in eukaryotic parasites, but also distinguish QS as a separate phenomenon from density dependent effects on infection dynamics. There is substantial literature examining how helminth abundance influences transmission to new hosts (*e.g.*, Anderson & May, 1985; Churcher, Ferguson & Basáñez, 2005; Neves et al., 2021), including dynamics specifically related to fish hosts (Poulin, 1998), but very little work addressing whether the strategies of individual parasites differ depending on the presence and density of conspecifics.

While coordinated egg hatching *via* QS is a plausible mechanism for increasing coinfection rates in copepods, followed by higher rates of outcrossing in birds, if and how *S. solidus* does this remains an open question. We therefore used laboratory-bred *S. solidus* to test whether egg density influences hatching rates, predicting that eggs in higher densities would also exhibit higher hatch rates, presumably mediated by chemical signals emitted by hatched worms (*i.e.,* a form of QS). Our experiment focused on detecting the species-level presence of density-dependent egg hatching. We additionally explored whether hatching rates varied by population and mating types (*i.e.,* outcrossed *versus* selfed progeny), though in both these cases our statistical inferences were limited by very small sample sizes. To date, no study has tested whether cestode egg hatching is density-dependent.

## Materials & Methods

### Egg collection

Portions of this text were previously published as part of a preprint (https://doi.org/10.1101/2025.07.02.662852). We collected *S. solidus*- infected threespine stickleback from Walby Lake, Alaska, USA (61.617963, −149.213644, ADFG permit number SF2022-043), Myvatn Lake, Iceland (65.596929, −17.002945), and Echo Lake, British Columbia, Canada (49.988258, −125.410090, BC Ministry of Forests permit number NA23-787881). Fish were captured using unbaited minnow traps left in the lake for no more than 10 h. After the traps were recovered, stickleback were housed in a bucket filled with lake water that was constantly aerated. As we held fish for less than one hour before euthanasia, no enrichment was provided. None of the fish displayed signs of severe distress during this holding period, such as inability to maintain orientation or buoyancy. Ten fish from each location were euthanized using an overdose of buffered MS-222, in compliance with the Institutional Animal Care and Use Committee of the University of Wisconsin–Madison (Protocol #L006460) and our experimental design followed ARRIVE guidelines. Vertebrates were not used for any other part of the study.

After euthanasia, we wrapped fish in damp paper towels, sealed them in plastic bags, and then transported chilled samples to our lab. Because the cestode remains alive in dead fish for up to a week, we were able to collect live parasites by dissecting fish under sterile lab conditions. We collected mature cestodes that had reached a size necessary to reproduce in bird hosts (*i.e.,* those with mass > 50 mg; Tierney & Crompton, 1992) and then followed a previously described *S. solidus* breeding protocol (Weber et al., 2017). A detailed protocol for *S. solidus* breeding, including product brands and relevant concentrations, is provided in File S1. Briefly, fertilized eggs were generated *via* three breeding designs: selfing of a single parasite, outcrossing between a pair of parasites with no more than 20 mg difference in mass, or a bulk outcross with six mature parents (<20 mg difference in mass). Size matching has been shown to increase the rate of outcrossing in *S. solidus* (Milinski, 2006). For all crosses, *S. solidus* were placed in sterile, nylon biopsy bags, which we flame-sealed to prevent escape. We then submerged the bags in a media containing HEPES buffering agent, L-Glutamine, antibiotic/antimycotic solution, and glucose to stimulate mating and release of gametes (Jakobsen et al., 2012; Smyth, 1946; Smyth, 1990; Weber et al., 2017). Nalgene wide mouth bottles containing media and cestodes were then placed into a shaking incubator at 42 °C and 50 rotations per minute. We inspected bottles daily to look for escaped worms and harvested eggs every two days. Bottles with escaped cestodes were excluded from the experiment. Egg production usually stopped by the 4–5th day of incubation.

### Density treatments

A total of 10 ml of Falcon tubes containing *S. solidus* eggs in RO water were gently inverted to create a uniform distribution of eggs in the solution. To generate variation in egg density, for each clutch we pipetted a range of egg solution (between 10 µl–500 µl) across wells in a 24-well plate, with between four-six replicate wells for each density (Table 1). RO water was added to each well to achieve a volume of ∼1ml. Plates were placed in a dark incubator at 18 °C for three days then, to induce hatching, were moved to room temperature and exposed to full spectrum light with an alternating light:dark cycle of 16 h:8 h (Scharsack, Koch & Hammerschmidt, 2007). In total, we made eight egg hatching plates (Table 1). The time between egg production and establishment of density treatments was not controlled, but each density plate was made within one year of the initial *S. solidus* crosses.

**Table 1:** Cestode egg clutches and populations used in the experiment. Echo Clutch 2, Myvatn Clutch 3 and Myvatn Clutch 4 were excluded from statistical analyses because they either produced few eggs or their hatching rates were too low to be accurately measured. Density replicates refers to how many wells on a 24-well plate were used for each density treatment. A single plate was used for each clutch.

Population	Clutch	Cross type	Density replicates	Egg densities (ul)
Walby	1	Bulk cross (6 parents)	6	100, 200, 400
	2	Paired outcross	4	10, 100, 200, 300, 400, 500
Myvatn	1	Paired outcross	6	100, 200, 400
	2	Paired outcross	4	50, 100, 200, 300, 400, 500
	3	Selfed	4	50, 100, 200, 300, 400, 500
	4	Selfed	4	50, 100, 200, 300, 400, 500
Echo	1	Bulk cross (6 parents)	4	50, 100, 200, 300, 400, 500
	2	Bulk cross (6 parents)	4	50, 100, 200, 300, 400, 500

### Measuring egg density and hatching rates

**Figure 1:**
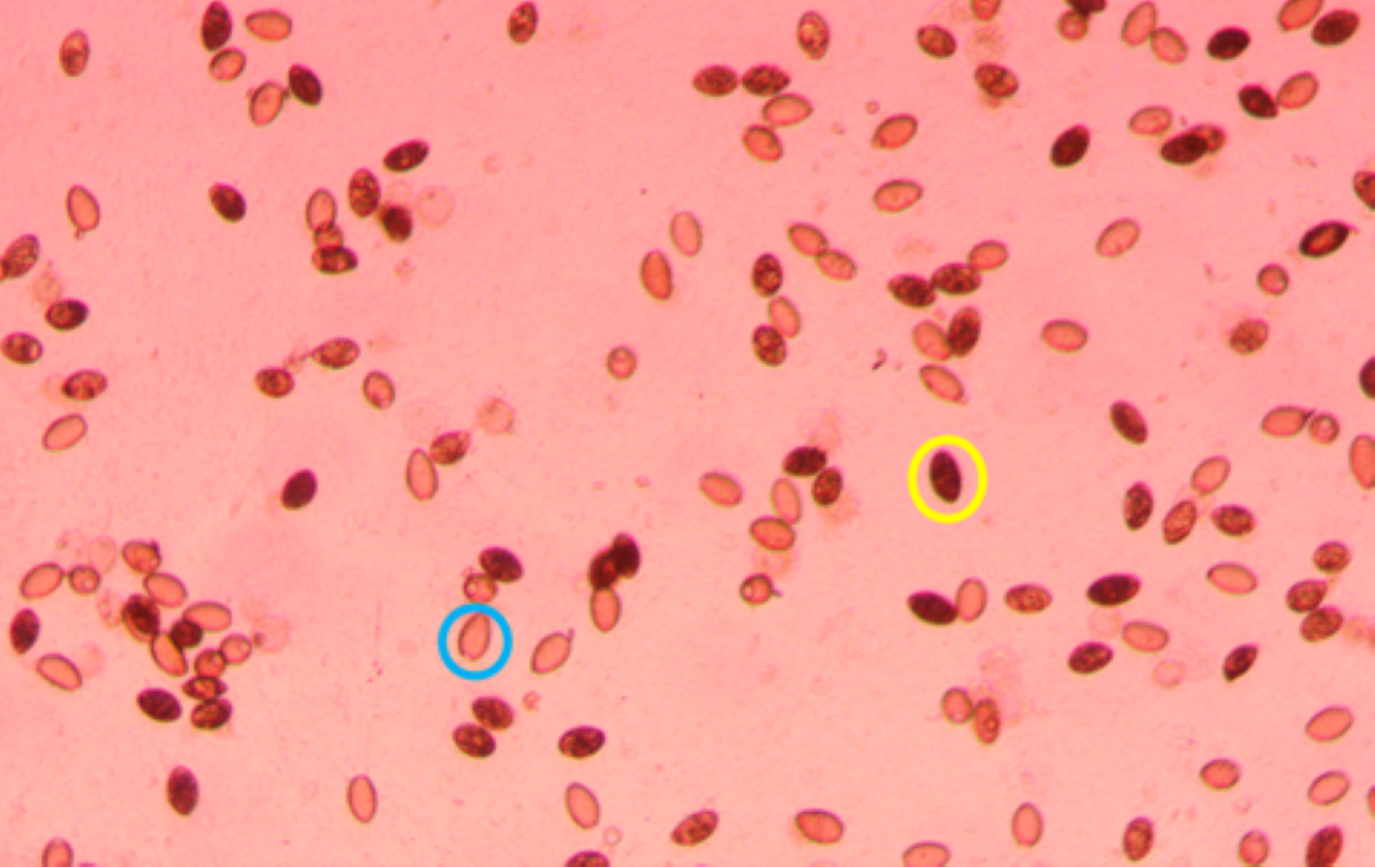
Portion of a photo (40x magnification) used to count *S. solidus* eggs, with an unhatched egg circled in yellow and a hatched egg circled in blue.

Plates were screened 2–3 times per week for hatching by scanning the water column for coracidia under a dissection microscope. Generally, hatching started ∼1 week after removal from the incubator and stopped 2–3 weeks later. After a week of observing no hatching, an inverted microscope was set to 40X and used to take a photo of each well. The location of each photo was decided by randomly selecting a location in the well when the microscope was not in focus. If the randomly selected location had less than 20 eggs in view, a new location was randomly chosen until the photo included at least 20 eggs. If all of the eggs were not in focus in a single photo, several photos were taken by first focusing on the eggs at the bottom of the water column and systematically moving to the top of the water column. If the photo was taken near the edge of the well, identical photos were taken with the brightness adjusted to ensure that all eggs were visible. After identifying the picture of each well with the most eggs in focus, two independent observers used QuPath-0.5.1 (Bankhead et al., 2017) to record the number of hatched and unhatched eggs (Fig. 1).

### Statistical analysis

All statistics were conducted in R (v. 4.3.2; R Core Team, 2023). To estimate the effects of experimental variables on egg numbers per well and hatch rates, we used linear mixed effects models implemented in the R package “lme4” (Bates et al., 2003). To determine if increasing egg volume led to linear increases in egg counts, we regressed log-transformed total number of eggs in each photo on clutch number, volume of egg solution, and their interaction. This model included observer as a random effect. The log transformation of egg counts was necessary to satisfy assumptions of normally distributed residuals in our linear regressions. We performed Spearman rank tests to correlate the predicted slope of clutch*volume interactions with egg density variation (*i.e.,* y-intercept of the model fits). We next tested for evidence of QS-mediated hatching. Because photos provided a coarse estimate of actual egg density in each well, we instead used the model estimate for log-transformed eggs in each clutch:volume combination for statistical analyses of hatching rate. We extracted model estimates of egg densities with the R package “car” (v. 3.1-2; Fox & Weisberg, 2019). We specifically modeled the percentage of hatched eggs as a function of ln(estimated egg density), including clutch number and observer as random effects. Our low sample sizes precluded further investigations of egg density effects. We next modeled the total hatch rate across populations, again using clutch number and observer as random effects. We used the R package “emmeans” (V. 1.10.3; Lenth, 2024) to compare population means, though we had too few samples to perform posthoc tests. We estimated *p*-values of all fixed terms in the mixed models *via* Type III Analysis of Variance with Satterthwaite’s method, implemented in R package “lmerTest” (v.3.1-3; Kuznetsova, Brockhoff & Christensen, 2017). We also anecdotally compared the effect of different cross types (*i.e.,* outcrossed pairs and bulk outcrosses) within a single population (Walby). The analysis script and raw data are available at https://github.com/EmilyKerns/SSolidusEggDensity. Photos of the eggs are available on Dryad.

## Results

Of the six examined outbred crosses, a single Echo plate failed to hatch. We occasionally observe crosses where egg hatching fails (J Weber, 2013–2025, pers. obs.), presumably due to lack of fertilization or damage to eggs during harvesting. As the failed Echo hatching was likely due to methodological errors, we excluded it from analyses. In contrast, while we did observe live coracidia when screening eggs from the two selfed crosses, the hatching rates were so low that hatched eggs were difficult to find at 40x magnification. Due to our inability to accurately measure hatching success, both selfed plates were also excluded from statistical analyses. This left us with a total of five plates for our analyses of variation in egg density and hatch rates.

**Figure 2:**
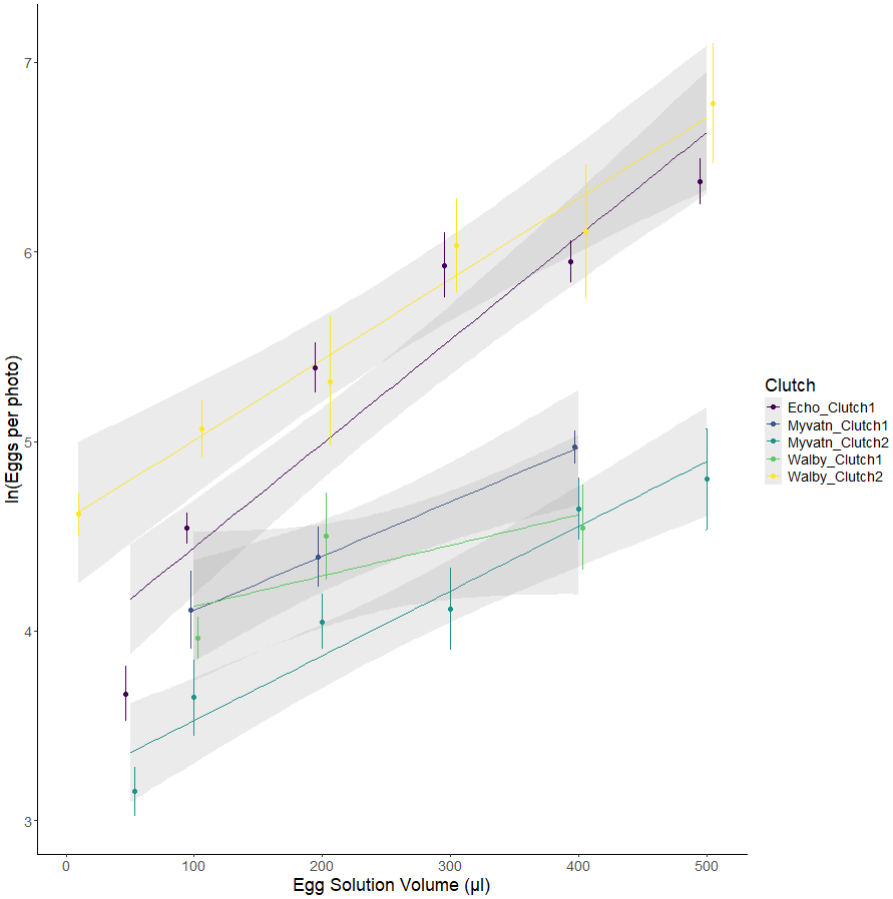
Observed variation in egg counts as a function of volume of egg solution used to seed the wells. Each color represents eggs generated from independent matings of cestodes from one of three geographic locations. Points represent the mean egg counts from replicate observations of each volume: clutch combination (±se). Lines represent the best fits of linear mixed effect models.

We initially tested whether our method for aliquoting eggs led to a predictable increase in egg density. We found significant additive effects of cross and egg volume, as well as a cross*volume interaction (Fig. 2; Table 2). Together, these factors accounted for the majority of variation in egg numbers across wells (R^2^_adj_ = 0.748). The slopes of egg number:volume associations should have increased with the density of eggs in solution; however, line slopes were not perfectly correlated with estimates of y-intercept (Spearman rho = 0.1, *p* = 0.95). This suggests that the significant interaction between clutch and volume may indicate differences in the degree to which eggs were homogenized prior to pipetting. Regardless, the strong positive association between egg volume and egg counts confirms that our experiment generated a range of egg densities for each clutch.

**Table 2:** Results of linear mixed models containing either interaction terms or only categorical fixed effects. Statistics represent Type III Analysis of Variance with Satterthwaite’s method. Significant predictors are in bold text.

Model	Predictor	Numerator DF	Denominator DF	*F*-value	*p*-value
ln(Total number of eggs)∼ Volume × Clutch + (1|Observer)	**Volume**	1	115	131.1583	**<0.001**
	**Cross**	4	115	7.5431	**<0.001**
	**Volume × Cross**	4	115	4.0140	**0.004**
Percent Hatched∼ Population + (1|Clutch) + (1 | Observer)	Population	2	2.000	16.389	0.058

**Figure 3:**
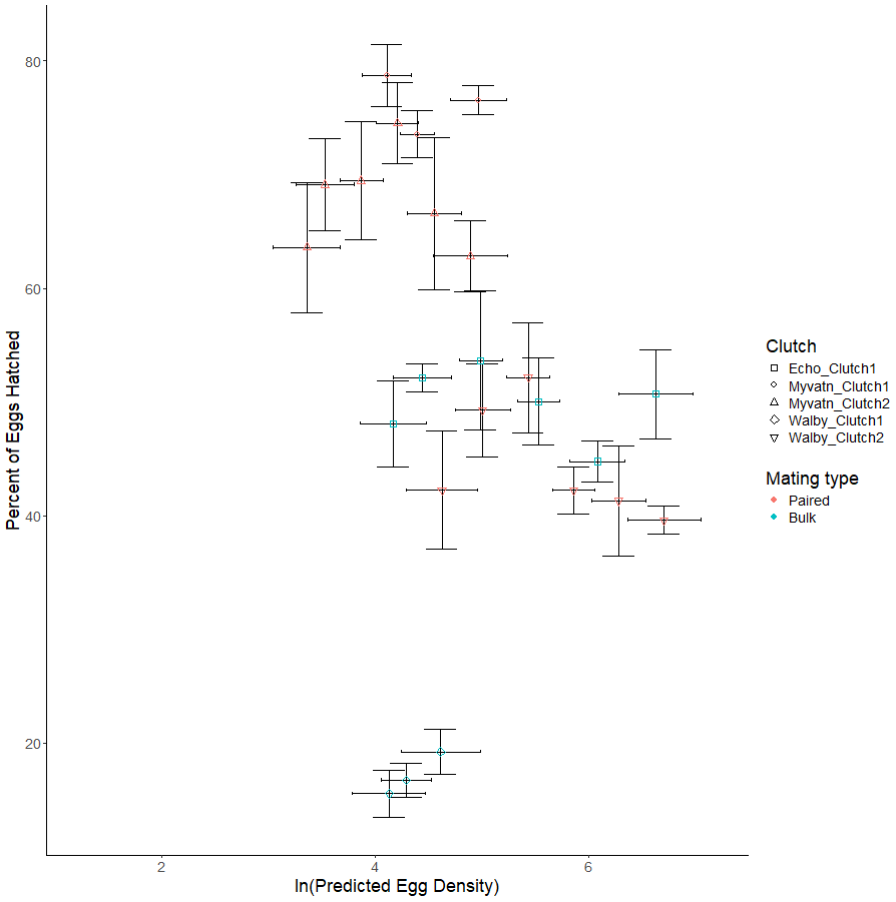
Observed variation in percent eggs hatched. Shapes represent independent egg clutches from one of three geographic locations. Points on the graph represent the mean percent of hatched eggs (±se) for predicted clutch-specific egg densities for each volume of egg solution. Horizontal error bars represent 95% confidence intervals for predicted egg density. Colors indicate whether eggs were generated *via* paired or bulk outcrosses.

We next considered whether increasing egg density generally increased hatching rates in all clutches, which was our main test of whether *S.solidus* displays QS. We found no effect of egg density on hatching rates (β = −1.299 ± 1.181, t_6.918_ = −0.2738, *p* = 0.275; Table 3, Fig. 3). Although we did not formally measure whether egg hatching varied spatially within each well, we can anecdotally report that hatching did not appear to be clustered within photos. Due to our limited sample sizes, we could not address whether hatching rate was influenced by interactions between egg density and other parameters. However, in an exploratory test we did find that parasite population was a predictor of hatching success (*F*_2,2_ = 16.389, *p* = 0.0575; Table 2; Fig. 3). Specifically, after averaging over outcross type, only 32% of Walby eggs hatched while rates in Echo and Myvatn eggs were respectively 50% and 81%. We had too few samples to perform posthoc analyses on these potential population differences in hatching rate.

**Table 3:** Result of linear mixed model regressing percent eggs hatched on predicted egg density. Statistics represent REML model fit and degrees of freedom (DF) estimated with Satterthwaite’s method.

Model	Predictor	Effect size	Std. Error	DF	*t*-value	*p*-value
Percent Hatched∼ ln(Predicted density) + (1|Clutch) + (1 | Observer)	ln(Predicted density)	−1.299	1.181	6.918	−0.2738	0.275

## Discussion

Previous work has shown that selfing in the cestode *S. solidus* greatly reduces the hatching rate of progeny (Benesh et al., 2014), suggesting that outcrossing should be preferred. However, *S. solidus* outcrossing primarily occurs when parasites encounter similarly sized mates (Milinski, 2006). This is presumably because of fitness tradeoffs associated with anisogamy, where a larger cestode has to invest disproportionately more energy into the production of large eggs that can be cheaply fertilized by the sperm of the smaller mate. Integrating these results led us to hypothesize that selection may act to coordinate the timing of hatching, thereby increasing the probability of parasites encountering similarly aged (and thus similarly sized) mates. We also hypothesized that if egg hatching is accompanied by a signal that can induce the hatching of other eggs, then hatching rates may increase at high egg densities. This would be similar to density-dependent signaling observed in other organisms (*i.e.,* QS; Coolahan & Whalen, 2025). To our knowledge, apart from the effects of outcrossing *versus* selfing, no experiments have examined factors that may influence variation in *S. solidus* egg hatching.

Although we were able to reliably generate and distribute *S. solidus* eggs across a range of densities, and also induce their hatching in our lab, we did not find evidence for density-dependent hatching (Fig. 3). This null result held both within and across three populations encompassing a broad geographic range. Although our low sample sizes provided little statistical power to interpret other variables affecting hatch rates, we did observe that three populations of parasites varied substantially in their hatching success: eggs from Lake Myvatn cestodes hatched nearly twice as often as those from Walby Lake. We could not formally compare hatching differences in cross type (*i.e.,* eggs produced from matings of six parents, two parents, or selfing) because these were confounded with population origin. However, we did observe extremely low hatch rates in two clutches of selfed eggs from Myvatn Lake cestodes. Specifically, across 48 observations of selfed eggs we observed less than 5 coracidia hatch and were unable to find any hatched eggs when looking under a microscope. We excluded these selfed clutches from our statistical analyses of percent eggs hatching because they would have effectively been 0% despite our knowing that some eggs did hatch. That said, given that over 80% of eggs hatched in the Myvatn paired outcrosses, our results support the expectation that a single selfing event has severe fitness costs in *S. solidus*.

Although we originally hypothesized that selection would favor QS in *S. solidus*, there are several lines of evidence that synchronized hatching and infection could have either neutral or deleterious effects. First, individual cestodes can vary greatly in their growth rates (Benessh & Hafer, 2012; Weber et al., 2017). Even if two cestodes infect either the same or different hosts at the same time, there is a reasonable chance they will differ greatly enough in size to preclude outcrossing. Previous studies also found that coinfection induces a crowding effect. Whether considering *S. solidus* infections in copepods (Wedekind, 1997) or threespine stickleback (Heins, Baker & Martin, 2002; Weber et al., 2017), there is negative correlation between infection intensity and parasite growth rate, which could lead to lower individual fecundity. Apart from direct competition between cestodes, coinfection can also stimulate greater immune responses within stickleback hosts. Specifically, some populations of stickleback have evolved the ability to encyst *S. solidus* in fibrotic tissue, thus preventing the cestode from growing and establishing an infection (Weber et al., 2017; Weber et al., 2022). Coinfection with multiple genotypes of *S. solidus* induces a higher fibrotic response than a single infection (Bolnick et al., 2024), increasing the risk of mortality for all cestodes within a fish. Finally, at least one study has found benefits of asynchronous infection (Jager & Schjørring, 2006). When two *S. solidus* were exposed to stickleback either synchronously or separated by one week, parasites in the later exposure had a significantly higher rate of infection.

While acknowledging that QS-based hatching may have a limited ability to increase outcrossing, or could even induce negative fitness effects, it is also instructive to consider whether there are particular conditions where it would be beneficial. One major assumption is that outcrossing is consistently beneficial in *S. solidus*. As noted previously, and in agreement with previous work (Benesh et al., 2014), we observed a fitness deficit associated with selfing. However, the outcrossing benefits of QS may depend on the relatedness of mates. The prevalence of *S. solidus* infection in stickleback varies drastically from lake-to-lake, with some populations having almost no infections (Weber et al., 2017). To our knowledge, only one study has examined the relatedness of *S. solidus* during coinfection (Sprehn et al., 2015). In this study, more than 90 percent of the total genetic variation segregated among cestodes within individual fish, suggesting that there is a high probability of encountering an unrelated mate. Interestingly, even in lakes with low infection prevalence, coordinated hatching that leads to coinfection with siblings (or closely related individuals) may still be beneficial. Specifically, outcrossing between siblings is predicted to be more efficient at purging inbreeding depression than selfing, at least during early generations of colonization (Porcher & Lande, 2016). This theoretical result begs a final consideration: should outcrossing remain beneficial after deleterious alleles are purged? *S. solidus* progeny produced after two consecutive generations of selfing had higher hatch rates than first-generation selfed progeny (Benesh et al., 2014). The authors of this study speculated that this increase might result from purging of deleterious alleles, and that most lethal alleles could be purged from selfed lines in as few as 10 generations. Therefore, the high cost of selfing may reduce over time, particularly in extremely isolated populations. Whether focusing on *S. solidus* or other hermaphroditic parasites, future experiments on the evolution of traits related to coinfection would benefit from comparing populations that differ in their heterozygosity or inbreeding coefficients. These genetic metrics could provide long-term summaries of mating outcomes, with the expectation that traits driving coinfection would be most beneficial in highly outbred populations.

Most of the data associated with QS come from bacterial studies, which could lead to an interpretation that this phenomenon is taxonomically restricted. However, the mechanistic features of QS are highly similar to a number of eukaryotic processes. For example, autocrine signaling among cells within an organism exhibits density-dependent properties (Doğaner, Yan & Youk, 2016), and numerous eukaryotes have evolved to recognize, respond to, and manipulate the QS signals of bacteria (Coolahan & Whalen, 2025). There is also strong evidence for density dependent signalling to conspecific eukaryotes. The parasite *Trypanosoma brucei*, which causes African sleeping sickness, uses QS to vary developmental outcomes and increase transmission rates (Matthews, 2021). Similarly, the fungal pathogen *Cryptococcus neoformans*, the most common cause of fungal meningitis in humans, evolved a QS mechanism that mediates several pathways associated with its virulence (Homer et al., 2016). Although there may indeed be taxonomic variation in the presence and use of QS, signals that coordinate the interactions of conspecifics merit additional research as they are largely neglected in ecological literature on positive density-dependent phenomena (Kramer, Berec & Drake, 2018; Stephens, Sutherland & Freckleton, 1990).

## Conclusions

QS is a common mechanism for coordinating positive density-dependent interactions in bacteria, but this mechanism is seldom examined in eukaryotes. We tested for the presence of QS-based egg hatching in the cestode *S. solidus,* using eggs from three geographically distant parasite populations. We found no evidence of QS as hatching rates did not correlate with egg concentrations, but the generalizability of our results are limited due to our small sample size. Despite this limitation, population origin did have a marginally significant effect on hatching success, and eggs from selfed matings hatched much less frequently than outcrossed eggs. Future studies on egg hatching rates in *S. solidus* and other helminths would benefit from not only testing whether population-specific hatch rates are common, but also addressing how population-specific differences in the benefits of synchronous infection or outcrossing may alter selection for QS-like traits.

## Supplemental Information

10.7717/peerj.20667/supp-1 Supplemental Information 1 Variation in egg numbers observed across all wells

10.7717/peerj.20667/supp-2 Supplemental Information 2 Weber Lab *Schistocephalus solidus* Breeding Protocol

10.7717/peerj.20667/supp-3 Supplemental Information 3 ARRIVE 2.0 checklist

## Additional Information and Declarations

### Field Study Permissions

The following information was supplied relating to field study approvals (i.e., approving body and any reference numbers): Cestodes are descendants of wild-caught individuals harvested from stickleback with permission from the Alaska Department of Fish and Game and British Columbia Ministry of Forests (ADFG Permit number: SF2022-043; BC Ministry of Forests Permit number: NA23-787881). No permit is required to sample stickleback or their parasites in Iceland.

### Data Availability

The following information was supplied regarding data availability: The code and raw data is available at GitHub and Zenodo: - https://github.com/EmilyKerns/SSolidusEggDensity. - EmilyKerns. (2025). EmilyKerns/SSolidusEggDensity: SSolidusEggDensity_121225 (1.0.0). Zenodo. https://doi.org/10.5281/zenodo.17914286
The data is available at Dryad:
https://datadryad.org/dataset/doi:10.5061/dryad.rbnzs7hqm.
