# Supplementary material for: No evidence for quorum sensing during egg hatching in the cestode Schistocephalus solidus
Source: PeerJ. 2026 Feb 3;14:e20667. doi: 10.7717/peerj.20667 (PMC12880099; doi:10.7717/peerj.20667)
Supplement: Supplemental Information 1 [file peerj-14-20667-s001.docx]

Supplementary Table 1: Variation in egg numbers observed across all wells.

| **Egg volume (ul)** | **Clutch** | **Well count** | **Mean** | **Std dev** | **Std Err** | **Max** | **Min** |
| --- | --- | --- | --- | --- | --- | --- | --- |
| 50 | Echo_Clutch1 | 4 | 40.5 | 12.8 | 6.4 | 59 | 31 |
| 100 | Echo_Clutch1 | 4 | 94.8 | 15.4 | 7.7 | 112 | 76 |
| 200 | Echo_Clutch1 | 4 | 225.0 | 60.7 | 30.4 | 308 | 162 |
| 300 | Echo_Clutch1 | 4 | 391.5 | 116.6 | 58.3 | 504 | 228 |
| 400 | Echo_Clutch1 | 4 | 391.5 | 92.2 | 46.1 | 521 | 321 |
| 500 | Echo_Clutch1 | 4 | 598.5 | 151.8 | 75.9 | 800 | 448 |
| 100 | Myvatn_Clutch1 | 12 | 73.9 | 44.7 | 12.9 | 185 | 11 |
| 200 | Myvatn_Clutch1 | 12 | 89.1 | 32.1 | 9.3 | 139 | 18 |
| 400 | Myvatn_Clutch1 | 12 | 150.1 | 43.7 | 12.6 | 216 | 87 |
| 50 | Myvatn_Clutch2 | 4 | 24.0 | 6.3 | 3.2 | 32 | 18 |
| 100 | Myvatn_Clutch2 | 4 | 40.5 | 14.4 | 7.2 | 55 | 23 |
| 200 | Myvatn_Clutch2 | 4 | 59.0 | 16.1 | 8.0 | 78 | 39 |
| 300 | Myvatn_Clutch2 | 4 | 65.3 | 23.6 | 11.8 | 88 | 33 |
| 400 | Myvatn_Clutch2 | 4 | 108.0 | 31.3 | 15.6 | 140 | 65 |
| 500 | Myvatn_Clutch2 | 4 | 134.5 | 67.2 | 33.6 | 220 | 63 |
| 100 | Walby_Clutch1 | 5 | 54.0 | 14.6 | 6.5 | 78 | 40 |
| 200 | Walby_Clutch1 | 6 | 103.8 | 67.3 | 27.5 | 231 | 52 |
| 400 | Walby_Clutch1 | 6 | 105.0 | 48.2 | 19.7 | 164 | 38 |
| 10 | Walby_Clutch2 | 4 | 103.0 | 24.7 | 12.4 | 140 | 89 |
| 100 | Walby_Clutch2 | 4 | 164.0 | 49.9 | 24.9 | 224 | 120 |
| 200 | Walby_Clutch2 | 4 | 240.3 | 148.1 | 74.0 | 397 | 105 |
| 300 | Walby_Clutch2 | 4 | 455.3 | 212.1 | 106.0 | 730 | 219 |
| 400 | Walby_Clutch2 | 4 | 522.5 | 266.8 | 133.4 | 769 | 160 |
| 500 | Walby_Clutch2 | 4 | 1007.0 | 517.7 | 258.8 | 1629 | 362 |
