# Supplementary material for: No evidence for quorum sensing during egg hatching in the cestode Schistocephalus solidus
Source: PeerJ. 2026 Feb 3;14:e20667. doi: 10.7717/peerj.20667 (PMC12880099; doi:10.7717/peerj.20667)
Supplement: Supplemental Information 2 [file peerj-14-20667-s002.docx]

**Weber Lab Schistocephalus solidus Breeding Protocol**

**Author: Jesse Weber**

**Updated: Cole Wolf, 9/18/23**

**Boil biopsy bags and fill/turn on the incubator before starting.**

Thaw frozen reagents in 42°C bath.

**Supplies**

- MEM medium, Sigma M2279 (500ml bottle)
- HEPES, sterile filtered, Sigma H0887, 25 ml/L (store at 4°C)
- L-Glutamine 200mM sterile filtered, Sigma G7513, 10mL/L (aliquot and store at -20°C)
- Antibiotic/Antimycotic 100x, sterile filtered, Sigma A5955, 10mL/L (aliquot and store at -20°C)
- Glucose stock, 325g/L distilled H20, autoclaved/filtered, 20ml/L (aliquot and store at -20°C)
- Bottles, Nalgene 250ml wide-mouth PPCO (**make sure autoclavable**), 2187-0008
  - To autoclave

1. Fill each bottle to top with distilled water
2. Close lid loosely and cover with foil and a piece of autoclave tape
3. Place in autoclave tray and fill bottom with ~1 inch of distilled water
4. Run on 15 min liquid autoclave cycle
5. Allow to cool and tighten caps. Can be stored filled with water and with foil on lid

- Nylon biopsy bags, Fisherbrand, Medium 45x75mm, 200 per box, 15-182-501 (note: smaller bags may be better for selfed crosses)
  - Preparation of biopsy bags

1. Boil for half an hour
2. Repeat twice
3. Autoclave in beaker covered with water
4. If reusing bags: autoclave in water; decant and store for future use in beaker covered with foil (water will evaporate on its own with time)

- Bunsen burner & lighter
- Long forceps
- Petri dishes
- 0.9x PBS (500ml 1x PBS + 55mL cell culture grade ddH20)
- Serological pipetman and pipettes
- Shaking incubator set to 42°C and speed of 50rpm.
- **Media recipe** (fills four breeding bottles, does not need to be made in hood, keep leftover media in fridge):
  - 1L MEM
  - 25ml HEPES
  - 10ml L-Glu
  - 10ml Antibiotic/Antimycotic
  - 20ml glucose

**Procedure**

1. Harvest worms from fish
   1. Dissect fish and transfer any worms to a petri dish containing 0.9x PBS. Be sure to label the base of the petri dish and not just the lid as the lids can get separated and easily mixed up.
   2. Record each worm on the datasheet. Be sure to record the lake origin of the fish and also assign each fish a unique number and each worm a letter.

Example data record:

| Lake | FIsh | Worm | Worm weight |
| --- | --- | --- | --- |
| Echo | 1 | a | 230mg |
|  | 1 | b | 120mg |
|  | 1 | c | 80mg |
| Echo | 2 | a | 210mg |
|  | 2 | b | 50mg |

- 1. Weigh each worm and record mass in notebook
  2. Pair worms for breeding based on mass (not visually by size)
  3. Record all cross information in notebook (Ex: Echo cross 1a x 2a)
  4. Update 2023: we typically make bulk crosses using the largest 6-10 worms from each population, as these produce a large number of eggs that hatch well.

1. Seal worms in bags
   1. Dampen bag with 0.9x PBS and insert worms gently using the flexible forceps. Try to place the worms in the very bottom of the bag so they can stay as far away from the burner as possible.
   2. Use a towel or kim wipe to dry off the side to be sealed.
   3. Hold at edge of bunsen burner flame with long forceps to seal. Sometimes I use a second pair of forceps to hold the near edge and make sure the worms don’t get close to the flame. It may take a minute or two for the PBS to evaporate and the bag to catch
   4. Insert top of bag with worms in the pipette groove attached to the cap of bottles. It helps to use forceps to pry open the groove and then insert the bag.
   5. Screw cap with worm bag attached into bottle with media. Leave foil on the lids. Ensure the bag is entirely submerged and add more media if needed.
   6. Label bottle with worm cross and place in a shaking incubator at 42C, shaking speed of 30rpm. Cover the incubators with a lid or cloth. Breeding needs to occur in total darkness.
   7. NOTE: Do not let water from the bath get into the breeding bottles - the water only needs to cover ~½ of the bottle. Remove some water from the water bath if necessary.
2. Check bottles daily for egg production and escaped worms
   1. Check every day by taking bottles out of the incubator and look for eggs (appears as dark powder) or escaped worms. Eggs should start appearing on the 2-3rd day.
   2. If eggs are present, transfer worms (with cap and everything) into a new labeled bottle with fresh media and put it back into the incubator. Note: the worms will usually stop producing eggs by the 4-5th day, so you shouldn’t have to do this more than 2-3 times.
   3. If worms have escaped, return the worms to the bag (or a new bag) and reseal. (note: if worms have been separated for a significant amount of time, they are likely to self instead of outcross)
3. Harvest, wash and store eggs
   1. Suction media out of the bottle using a vacuum tube connected to a long glass pasteur pipette. Remove as much media as possible without sucking out eggs. It is fine to use the same pipette for all bottles as long as you suction clean water between samples.
   2. Wash eggs with autoclaved (or filtered) distilled water. Fill the bottle with water and let eggs settle for ~30 min-1 hour
   3. Aspirate water being careful not to disturb the eggs.
   4. Repeat water washes 2-3 times, until water is no longer pink.
   5. After final water change, and aspirating almost all the water out, transfer eggs into 15ml conical tubes using the serological pipetman. Fill the 15ml conical tube all the way to the top with autoclaved distilled water.
   6. Label both the tube and lid (use the label stickers to make this easier!). Cover in foil and place labels on the outside of foil as well. Store the tubes upright in a box or beaker, so that eggs pellet at the bottom of the tube. **Be sure the label includes all cross information and date.**
   7. Depending on the number of crosses, you may need to wash and autoclave nalgene bottles and more distilled water as you go.
   8. If there are still eggs being produced at day 5, collect them. However most crosses are exhausted by this point. If only a few eggs are being produced, they likely are not worth washing and saving, can be discarded along with the media.
   9. If DNA from parents is wanted, store worms in EtOH in labeled Eppendorf 1.5ml tubes and freeze at -20C. Otherwise discard worms and media.
